# Corticobasal syndrome and Parkinson’s disease at the beginning: asymmetrical patterns of MRI and Blink Reflex for early diagnosis

**DOI:** 10.1007/s00702-022-02557-7

**Published:** 2022-10-29

**Authors:** Giulia Donzuso, Giorgia Sciacca, Antonina Luca, Calogero E. Cicero, Giovanni Mostile, Alessandra Nicoletti, Mario Zappia

**Affiliations:** Department of Medical, Surgical Sciences and Advanced Technologies “GF Ingrassia”, University of Catania, Via Santa Sofia 78, 95123 Catania, Italy

**Keywords:** Parkinson’s disease, Corticobasal syndrome, Diagnosis, Blink reflex, Magnetic resonance imaging

## Abstract

Differential diagnosis between Parkinson’s disease (PD) and corticobasal syndrome (CBS) could be challenging at the early stage, due to the asymmetric onset of both diseases. Despite the clinical overlap, the anatomical circuits involved in these disorders are different. We evaluated R2 Blink Reflex Recovery Cycle (R2BRRC) and cortical thickness (CTh) in drug-naïve PD and CBS patients for characterizing pathophysiological mechanisms underlying these conditions. Patients with a clinically probable diagnosis of PD and possible CBS were recruited. R2BRRC was evaluated bilaterally at interstimulus intervals (ISIs) of 100–150–200–300–400–500–750 ms. Asymmetry index (AI) of R2BRRC for each ISI was computed. Patients underwent a structural brain MRI and hemisphere CTh and AI of MRI was calculated. Fourteen drug-naïve PD patients and 10 patients with early CBS diagnosis were enrolled. R2BRRC of PD patients showed an increased brainstem excitability for less affected side (LAS) stimulation at ISIs of 100 and 150 ms (*p* < 0.001) compared to most affected side (MAS), whereas no differences between LAS and MAS were found in CBS. AI of R2BRRC at ISI-100 ms showed significant difference, being higher in PD. CTh analysis showed significant differences between groups in hemisphere cortical volume contralateral to MAS, and, conversely, AI of MRI was significantly higher in CBS. PD patients exhibited an asymmetric pattern of brainstem excitability, compared to CBS. Conversely, CBS patients showed an asymmetric pattern of cortical atrophy. This opposite pattern of neurophysiological and structural abnormalities involving cortical and subcortical brain structures could highlight the different pathophysiological mechanisms underlying these disorders.

**Supplementary Information:**

The online version contains supplementary material available at 10.1007/s00702-022-02557-7.

## Introduction

Atypical parkinsonian syndromes (APs), such as corticobasal syndrome (CBS), are rare neurodegenerative disorders, characterized by a more rapidly progression and a poor levodopa responsiveness in comparison with Parkinson’s disease (PD) (Jabbari et al. 2020). Nevertheless, clinical differential diagnosis between CBS and PD could be challenging, especially at the beginning of the disease, because of common clinical features and the asymmetric onset of the disorders.

R2 Blink Reflex Recovery Cycle (R2BRRC) is a neurophysiological tool used to assess brainstem excitability, typically increased in patients affected by PD (Kimura 1973). In particular, the asymmetry index (AI) of R2BRRC differentiates early PD patients from patients affected by multiple system atrophy (MSA) and progressive supranuclear palsy (PSP) with a specificity of 100% (Sciacca et al. 2020a). On the other hand, the use of optimal R2BRRC cutoff scores could be used in differential diagnosis of CBS from MSA and PSP in daily clinical practice, with CBS patients showing a normal brainstem excitability (Sciacca et al. 2018, 2020b).

Considering neuroimaging studies, including structural magnetic resonance imaging (MRI), CBS patients typically present an asymmetrical cortical atrophy, involving posterior frontal and parietal lobes, contralateral to the clinically most affected side (MAS) (Constantinides et al. 2019). In addition, CBS showed a more prominent supratentorial pattern of atrophy in comparison with other APs, such as PSP patients, having an infratentorial involvement and symmetric pattern (Whitwell et al. 2014). However, at the early stages of the disease, qualitative evaluation of MRI could not be conclusive for differential diagnosis between PD and CBS. Thus, the use of advanced MRI neuroimaging method with quantitative analysis of cortical structures together with neurophysiological data exploring brainstem excitability could be useful for the differential diagnosis.

The aim of this exploratory study is to evaluate brainstem excitability by AI of R2BRRC and hemisphere cortical thickness volume by the computation of an AI of MRI, in drug-naïve PD and CBS patients for helping in differential diagnosis at the early stage of diseases.

## Methods

### Patients and clinical assessment

Drug-naïve patients affected by PD and patients with early CBS who met diagnostic criteria for clinically probable PD and possible CBS, respectively, were enrolled according to currently accepted diagnostic criteria (Postuma et al. 2015; Armstrong et al. 2013). Clinical diagnosis of PD and CBS was confirmed by a subsequent 2-year clinical follow-up. The study was approved by the Local Ethics Committee and patients were enrolled after signing the written informed consent. None of the patients was under treatment with anticholinergic agents, antidepressants, or other centrally acting drugs. Clinical evaluation was performed through Unified Parkinson’s Disease Rating Scale—Motor Examination section (UPDRS-ME) (Fahn et al. 1987) and Hoehn and Yahr (H&Y) stage (Hoehn and Yahr 1967). More affected side (MAS) compared to less affected side (LAS) was defined as more clinical impaired side at the time of enrollment, evaluating the presence of tremor, rigidity, and bradykinesia through UPDRS-ME scale between the two sides of the body. The subscores of UPDRS-ME scale have been calculated from the item 20–26 to objectively differentiate MAS from LAS for each group of patients.

### Neurophysiological evaluation

Blink reflex (BR) and R2BRRC were recorded in all patients by a neurophysiologist unaware of clinical data. A bipolar electrical stimulation was applied to supraorbital nerve (intensity: 15–25 mA; duration: 0.2 ms) and electromyographic responses were recorded in orbicularis oculi muscles through surface silver–silver chloride electrodes (filters: 20 Hz–10 kHz) (Kimura 1973). R2BRRC was performed with the technique of paired stimulation at interstimulus intervals (ISIs) of 100, 150, 200, 300, 400, 500, and 750 ms. For each ISI the R2 amplitude ratio (expressed as percentage ratio between R2 peak-to-peak amplitudes of conditioned and unconditioned responses) was calculated (Kimura 1973). R2BRRC was evaluated by plotting the R2 amplitude ratio for all the tested ISIs. As previously described (Sciacca et al. 2020a), the absolute value of AI of R2BRRC was estimated using the following formula: [(Side1 − Side2)⁄(Side1 + Side2)], where the two sides are the percentage values of R2BRRC for each ISI, calculated by stimulating both MAS and LAS of each patient.

### MRI data acquisition

Brain MRI was performed according to our routine protocol with a 1.5 T unit (Signa HDxt, GE Medical Systems, Milwaukee, WI, USA). A 3D T1-weighted high-resolution spoiled gradient echo (SPGR) sequence with a 1.2-mm slice thickness and an isotropic in-plane resolution of 0.98 mm was acquired with the following parameters: repetition time 14.8 ms, echo time 6.4 ms, flip angle 25°, 115 slices, matrix size 256 X 256 and a field of view of 24 cm. In addition, all the patients underwent also a T2-weighted and FLAIR images in order to exclude morphological abnormalities, vascular disease or intracranial lesions.

### Image analysis

Cortical reconstruction was performed using the FreeSurfer image analysis suite, version 5.3, documented and freely available for download online (http://surfer.nmr.mgh.harvard.edu/). Briefly, this processing includes motion correction and averaging of multiple volumetric T1-weighted images, removal of non-brain tissue using a hybrid watershed/surface deformation procedure, automated Talairach transformation (Fischl et al. 2004), intensity normalization, tessellation of the gray-white matter boundary, automated topology correction and surface deformation following intensity gradients, to optimally place the gray/white and gray/cerebrospinal fluid borders at the location where the greatest shift in intensity defines the transition to the other tissue class (Fischl and Dale 2000). Images are then carefully checked for skull stripping errors. Once the cortical models are complete, a number of deformable procedures can be performed for further data processing and analysis including surface inflation, registration to a spherical atlas which is based on individual cortical folding patterns to match cortical geometry across subjects, parcellation of the cerebral cortex into units with respect to gyral and sulcal structure (Desikan et al. 2006), and creation of a variety of surface-based data including maps of curvature and sulcal depth. This method uses both intensity and continuity information from the entire three-dimensional MR volume in segmentation and deformation procedures to produce representations of cortical thickness, calculated as the closest distance from the gray/white boundary to the gray/CSF boundary at each vertex on the tessellated surface (Fischl and Dale 2000). Individual surface maps are registered to a common average surface and then smoothed using a Gaussian kernel of 10 mm full width half-maximum.

Mean hemisphere cortical thickness volume for each patient was obtained and then attributed to the clinical MAS and LAS. To compute the AI of MRI, we considered global mean cortical thickness of each hemisphere contralateral to the clinical MAS and LAS and applied the following formula: [(Side1 − Side2)⁄(Side1 + Side2)], where the two sides are the mean cortical thickness values for MAS and LAS of each patient.

### Statistical analysis

Data were analyzed using STATA 12.1 software packages (StataCorp, College Station, TX, USA) and easyROC (version 1.3.1; www.biosoft.hacettepe.edu.tr/easyROC). Quantitative variables were described using mean and standard deviation. Differences between means and proportions were evaluated by *t* test and the Chi-square test, respectively. In case of not normal distribution, appropriate non-parametric tests were performed. For each AI, the absolute value ranged between 0 and 1, where 0 represented the absence of asymmetry and 1 represented the maximum asymmetry between the two sides. A receiver-operating characteristic (ROC) curve was developed, obtaining area under the curve (AUC) and cutoff values with Youden optimal cutoff method for each AI, and for a combination of both AI of R2BRRC and AI of MRI. In addition, a logistic regression analysis was conducted for testing the association between AIs and diagnosis.

## Results

### Clinical and neurophysiological data

**Fig. 1:**
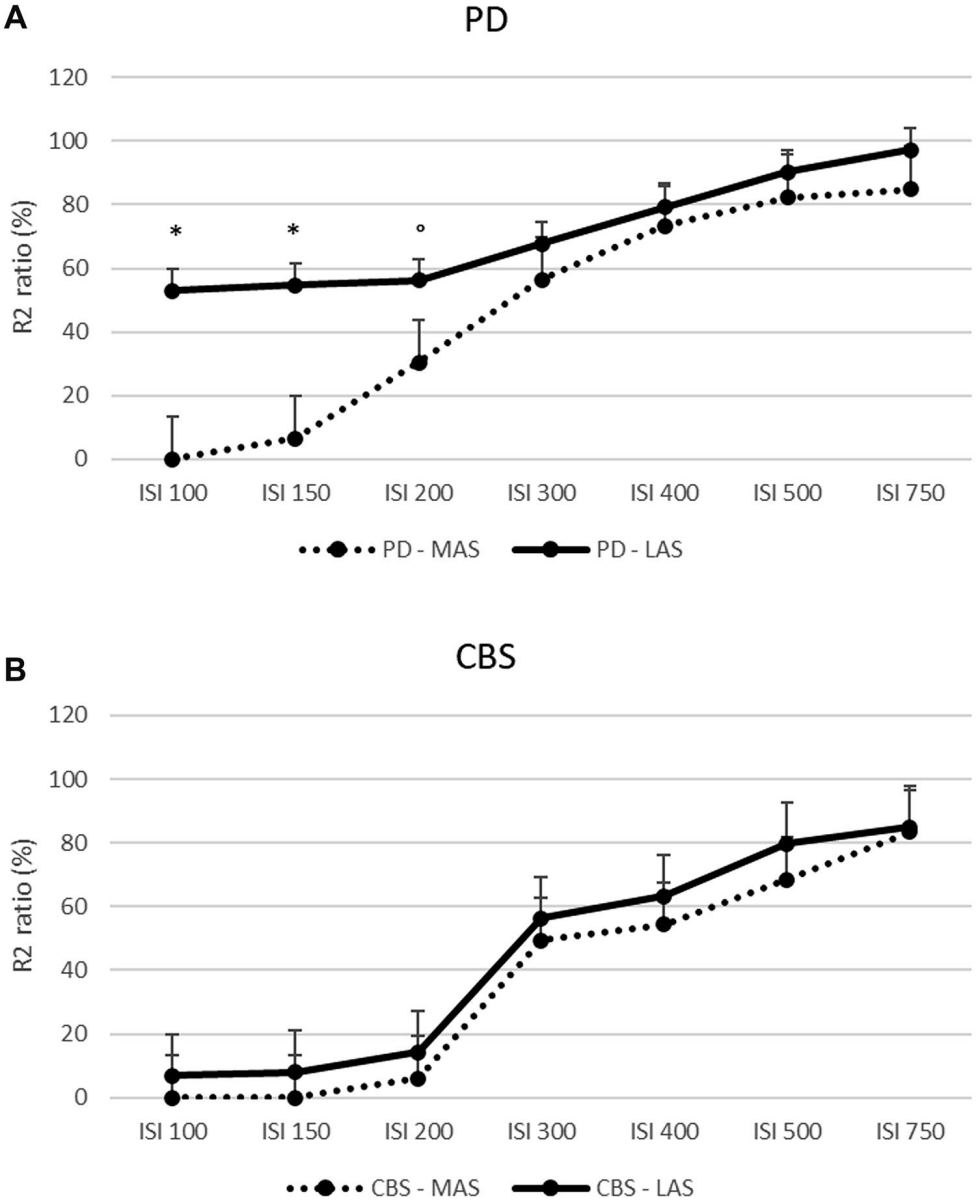
R2 Blink Reflex Recovery Cycle graph-curve for PD and CBS patients. Ratios of the conditioned R2 component (amplitude) to the unconditioned response are shown as mean + standard error (S.E.). X-axis: interstimulus intervals (ISIs) in milliseconds (ms). Y-axis: ratio of the conditioned to the unconditioned R2 response in percentage (%). **A** R2 Blink Reflex Recovery Cycle graph-curve of PD patients; paired *t* test: *p < 0.001, °*p* = 0.02 when comparing more affected side (MAS) stimulation vs. less affected side (LAS) stimulation. **B** R2 Blink Reflex Recovery Cycle graph-curve of CBS patients; no statistically significant differences were found when comparing MAS stimulation vs. LAS stimulation. *PD* Parkinson’s disease, *CBS* corticobasal syndrome

**Fig. 2:**
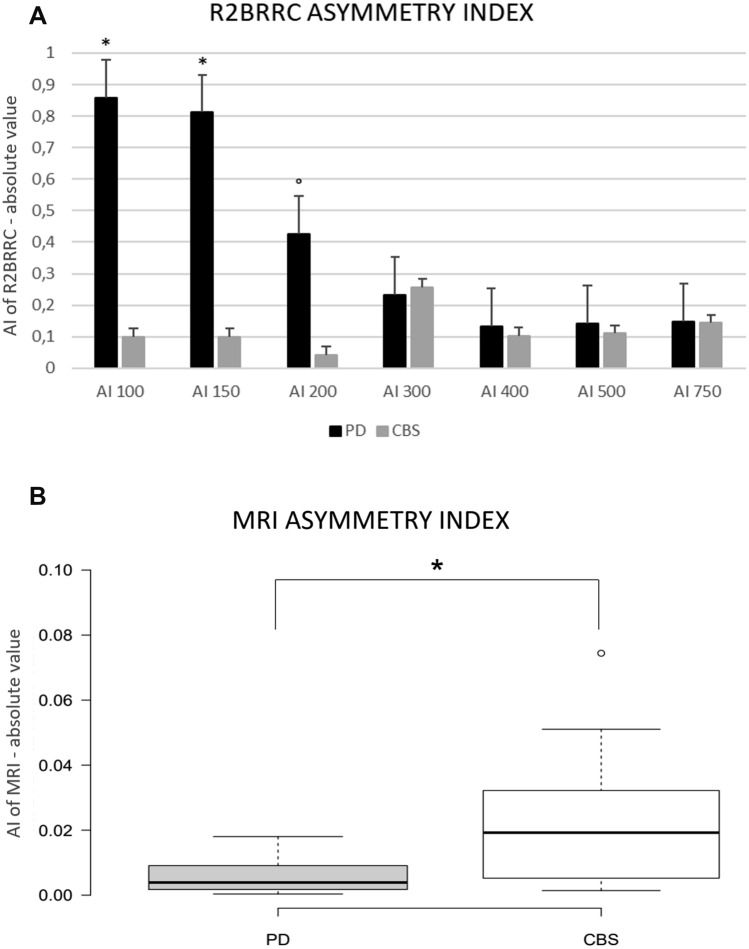
Asymmetry indexes for patients affected by Parkinson’s disease and corticobasal syndrome. Asymmetry indexes are shown as mean + standard error (bars). **A** AI of R2BRRC. *p < 0.001 and °*p* = 0.004 when comparing PD and CBS; **B** AI of MRI. *p = 0.01. Outliers are represented by dots. *PD* Parkinson’s disease, *CBS* corticobasal syndrome

Fourteen drug-naïve PD patients and 10 patients with early CBS diagnosis were enrolled. PD patients were younger and had a lower UPDRS-ME score than CBS patients (Table 1). After a 2-year period, all PD and CBS patients met their respective diagnostic criteria, confirming clinically probable PD and possible CBS. R2BRRC of PD patients showed an increased brainstem excitability for LAS stimulation at ISIs of 100, 150 (*p* < 0.001) and 200 ms (*p* = 0.02) compared to MAS (Fig. 1A), whereas no differences between LAS and MAS were found in CBS (Fig. 1B). AI of R2BRRC at ISI of 100, 150 and 200 ms showed significant difference between groups, being higher in PD (Table 2 and Fig. 2A). Among CBS patients, only one showed atypical value (marked asymmetry) for AI of R2BRRC, while in the PD group, two patients exhibited no asymmetry of AI of R2BRRC. Multivariate analysis adjusted for age showed a significant association between diagnosis and AI of R2BRRC, with *p* = 0.02.

**Table 1:** Demographics and clinical characteristics of patients affected by Parkinson’s disease and corticobasal syndrome

	PD	CBS	*P* value
	*n* = 14	*n* = 10	
Gender (men)^§^	7 (50.0%)	4 (40.0%)	0.39
Age (years)^	64.6 ± 7.5	71.1 ± 5.9	*0.03*
Disease duration (years)^	1.7 ± 1.3	2.9 ± 1.4	0.07
UPDRS-ME score^	24.8 ± 11.8	37.6 ± 15.2	*0.03*
HY stage^	1.9 ± 0.3	2.1 ± 0.5	0.1
Clinical MAS (right)^§^	7 (50%)	7 (70%)	0.9

Data are given as means ± standard deviations^ or number of subjects (%)^§^

*PD* Parkinson’s disease, *CBS* corticobasal syndrome, *UPDRS-ME* Unified Parkinson’s Disease rating scale—motor examination, *HY* Hoehn–Yahr stage, *MAS* more affected side

**Table 2:** Asymmetry indexes of R2 Blink Reflex Recovery Cycle and MRI data in patients with Parkinson’s disease and Corticobasal syndrome

	PD	CBS	*P* value
	*n* = 14	*n* = 10	
AI of R2BRRC			
ISI 100	0.86 ± 0.36	0.10 ± 0.32	*0.0001*
ISI 150	0.81 ± 0.37	0.10 ± 0.32	*0.0003*
ISI 200	0.42 ± 0.36	0.04 ± 0.10	*0.004*
MRI data			
Total GM volume (mm^3^)	402,387 ± 28,257	346,242 ± 24,986	*0.0002*
MRI MAS volume (mm^3^)	200,090 ± 14,971	171,191 ± 9009	< *0.0001*
AI of MRI	0.006 ± 0.005	0.02 ± 0.02	*0.01*

Data are given as means ± standard deviations

*PD* Parkinson’s disease, *CBS* corticobasal syndrome. *AI* of *R2BRRC* asymmetry index of R2 Blink Reflex Recovery Cycle. *GM* grey matter, *MRI* magnetic resonance imaging, *MAS* more affected side, AI of MRI asymmetry index of magnetic resonance imaging

AI of R2BRRC cutoff greater than 0.75 (accuracy: AUC = 0.94; *p* < 0.001), differentiated drug-naïve PD from CBS patients with a sensitivity of 85.7% (95% CI 57.2–98.2) and a specificity of 90.0% (95% CI 55.5–99.7) (figure S1).

### MRI data

Cortical thickness analysis showed significant differences between groups in total GM volume and in hemisphere volume considering MRI MAS. In addition, AI of MRI was significantly higher in CBS group (Table 2 and Fig. 2B).

AI of MRI cutoff greater than 0.014 (accuracy: AUC = 0.77; *p* = 0.002), differentiated CBS from drug-naïve PD patients with a sensitivity of 70% (95% CI 34.8–93.3) and a specificity of 85.7% (95% CI 57.2–98.2) (figure S1). Combination of the two AIs showed an AUC of 1.0, with a sensitivity and specificity of 100% in differentiating PD and CBS patients (figure S2).

## Discussion

In the present study, we try to provide some instrumental tools to distinguish PD and CBS in their early phases. As previously demonstrated, drug-naïve PD patients exhibited an increased brainstem excitability contralateral to clinically affected side by R2BRRC and higher AI of R2BRRC compared to CBS. Conversely, CBS patients did not show any increased excitability between MAS and LAS neither asymmetric pattern of brainstem excitability compared to PD patients considering AI of R2BRRC. Moreover, considering structural MRI data, CBS patients exhibited a decrease total GM and hemispheric cortical thickness volume contralateral to clinical MAS, and on the contrary, showed a higher AI of MRI in comparison with PD patients.

Previous neurophysiological studies evaluated R2BRRC in PD and APs showing that PD patients exhibited increased brainstem excitability (Kimura 1973; Sciacca et al. 2020a); furthermore, an asymmetrical EMG activity with increase of blink reflex components on the LAS compared to MAS has been observed in PD patients (Dengler et al. 1982) together with the presence of a higher AI of R2BRRC differentiating PD from PSP and MSA patients (Sciacca et al. 2020a). Concerning APs, only a few studies investigated brainstem excitability by the application of blink reflex and R2BRRC (Sciacca et al. 2020a, b; Sciacca et al. 2018; Valls-Solé et al. 1997; Szmidt-Salkowska et al. 2016) showing that PSP and MSA patients exhibited an increased brainstem excitability compared to CBS patients. In this study, we added and confirmed previous data showing that PD and CBS patients at the early stages of disease presented an inverse pattern of brainstem excitability, with PD patients showing an increased excitability in LAS compared to MAS and a higher AI of R2BRRC, whereas CBS group did not show any differences between LAS and MAS excitability.

Considering MRI findings, cortical thickness findings demonstrated that CBS patients had a decrease total and hemisphere GM volume considering MRI MAS and exhibited a higher AI of MRI. Structural MRI studies showed that CBS patients had a typical asymmetrical pattern of cortical atrophy involving fronto-parietal areas (Constantinides et al. 2019) with a prominent supratentorial involvement (Whitwell et al. 2014), and a relatively preserved brainstem anatomy (Kitagaki et al. 2000). In accordance with our findings, Kitagaki et al. (2000) showed that CBS patients had a widespread cortical atrophy involving the whole cortex and a higher hemispheric asymmetric index compared to AD patients (Kitagaki et al. 2000). Again, CBS patients exhibited a decreased cortical thickness compared to healthy controls, an asymmetric pattern of cortical atrophy with a thinner hemisphere contralateral to the MAS, a sparing of subcortical structures (Upadhyay et al. 2016) and a preservation of brainstem structures (Bocchetta et al. 2020). On the other hand, it has been demonstrated that PD patients did not show an asymmetric cortical atrophy despite the presence of an asymmetric clinical presentation (Danti et al. 2015).

Together with neurophysiological and structural MRI data, few functional MRI studies on CBS showed an increased connectivity in cortical areas, including sensorimotor and executive-control network, and abnormal connectivity between frontal lobe and thalamus, probably related to the underlying tau burden (Spina et al. 2019; Upadhyay et al. 2017) without brainstem involvement, while it has been widely demonstrated the abnormal cortico-subcortical functional connectivity in PD patients, mainly involving basal ganglia and midbrain (Filippi et al. 2019).

Some limitations and weaknesses of our study should be addressed. First, despite diagnostic criteria strongly accepted have been applied, and a 2-year clinical follow-up has been conducted, our study does not address the issue of the pathology underlying the CBS, thus the lack of pathological confirmation of the diagnosis could determine a diagnostic selection bias. It is well known that corticobasal degeneration (CBD) is considered a tauopathy, together with AD, PSP and frontotemporal dementia (FTD) and that pathological features such as ballooned or achromatic neurons in cortical GM, or astrocytic plaques were considered pathognomonic of CBD (Constantinides et al. 2019). PET imaging could help to differentiate AD and tau pathology, but unfortunately, amyloid and tau PET were not available; therefore, we cannot exclude that our findings would have been different if a non-CBD pathology was present in our CBS patients.

Furthermore, the conceptual problem in comparing different parkinsonian syndromes based on clinical diagnostic criteria still remains. PD diagnosis is reconsidered up to 30% of the patients during the follow-up (Coarelli et al. 2019) and a diagnostic switch to other causes of parkinsonism, specifically CBS, could occur in 3.6% of cases (Keshtkarjahromi et al. 2022). In our study design, the enrolled patients fulfill an initial diagnosis based on currently accepted clinical diagnostic criteria (clinically probable PD and possible CBS). We are aware of the limitation due to a possible diagnostic bias, as above discussed, but in this context, the availability of simple and reproducible measurements, such as those proposed in our study could be useful for differential diagnosis especially at the early stages of the diseases.

Second, the small sample size, considering the low frequency of CBS, justified the exploratory nature of the study. Thus, longitudinal and multicentric studies on larger populations are needed. On the other hand, even if exploratory, we provide some new insights in addressing the early differentiation of these conditions.

In conclusion, we hypothesized that the different and asymmetrical neurophysiological and brain structural pattern observed in these patients’ groups could reflect the different anatomopathological features in PD and CBS, with the major involvement of brainstem and neocortex, respectively, affecting pathophysiological mechanisms underlying these two neurodegenerative disorders. Thus, the combination of the two AIs could be a useful clinical tool helping clinicians in differentiating PD and CBS patients at the beginning of the disease. Alongside, the importance of identifying structural and/or functional features helping in differentiating and predicting disease progression or therapy response (Contrafatto et al. 2011) could be useful in consideration of the principles of precision medicine.

## Supplementary Information

Below is the link to the electronic supplementary material.

Supplementary file1 (DOCX 248 KB)

## Data Availability

Anonymized data will be made available on reasonable request.
