# Supplementary material for: Corticobasal syndrome and Parkinson’s disease at the beginning: asymmetrical patterns of MRI and Blink Reflex for early diagnosis
Source: J Neural Transm (Vienna). 2022 Oct 29;129(12):1427–33. doi: 10.1007/s00702-022-02557-7 (PMC9649477; doi:10.1007/s00702-022-02557-7)
Supplement: Supplementary file 1 — Supplementary file1 (DOCX 248 KB) [file 702_2022_2557_MOESM1_ESM.docx]

**Supplementary materials**

**Supplementary figure 1.** ROC curve for AIs of R2 blink Reflex Recovery Cycle and MRI data.


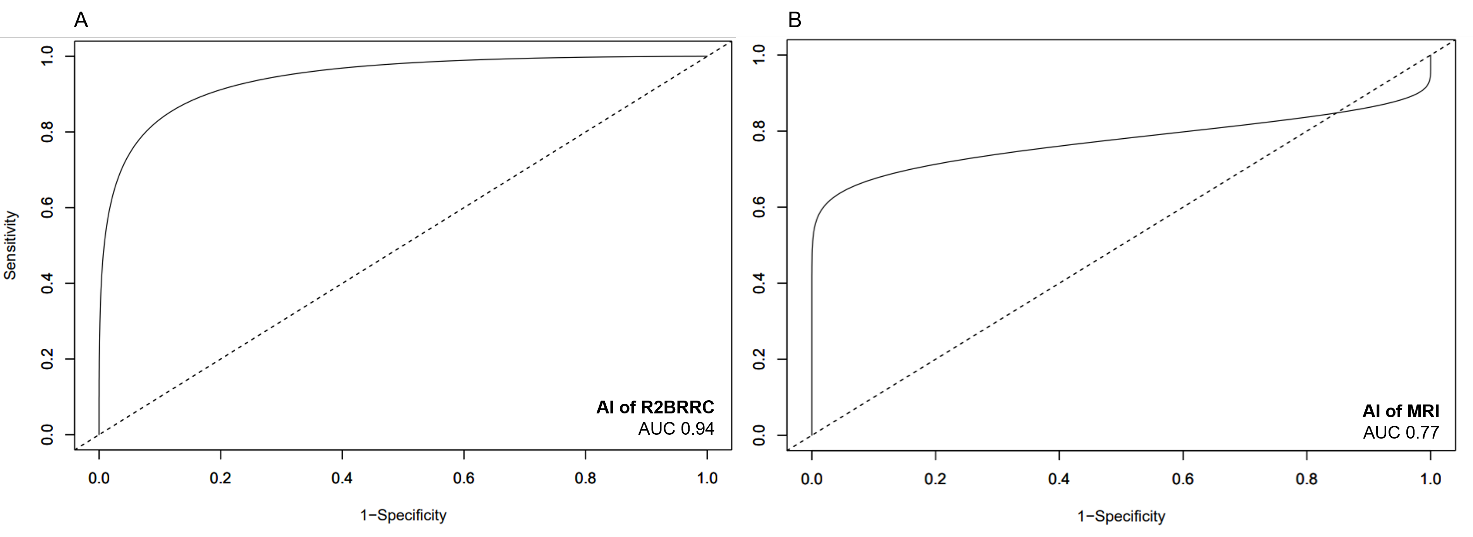


A. ROC curve for AI of R2BRRC: cut-off greater than 0.75 (accuracy: AUC=0.94; p<0.001), sensitivity 85.7%, specificity 90.0%.

B. ROC curve for AI of MRI: cut-off 0.014 (accuracy: AUC=0.77; p=0.002), sensitivity 70%, specificity of 85.7%.

ROC, receiver operating characteristic; AI of R2BRRC, Asymmetry Index of R2 blink Reflex Recovery Cycle. AI of MRI, Asymmetry Index of Magnetic Resonance Imaging.

**Supplementary figure 2.** ROC curve for combinational analysis of AIs of R2 blink Reflex Recovery Cycle and MRI data.


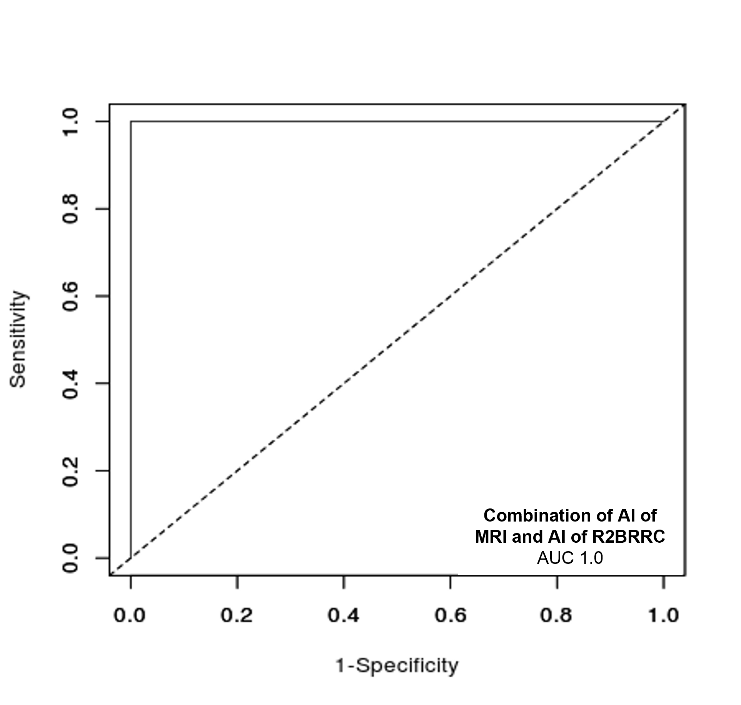


ROC curve for AI of R2BRRC and AI of MRI: sensitivity 100%, specificity 100%.

ROC, receiver operating characteristic; AI of R2BRRC, Asymmetry Index of R2 blink Reflex Recovery Cycle. AI of MRI, Asymmetry Index of Magnetic Resonance Imaging.
